# Systemic Acquired Resistance-Mediated Control of Pine Wilt Disease by Foliar Application With Methyl Salicylate

**DOI:** 10.3389/fpls.2021.812414

**Published:** 2022-01-05

**Authors:** Hee Won Jeon, Ae Ran Park, Minjeong Sung, Namgyu Kim, Mohamed Mannaa, Gil Han, Junheon Kim, Yeonjong Koo, Young-Su Seo, Jin-Cheol Kim

**Affiliations:** ^1^Department of Agricultural Chemistry, College of Agriculture and Life Sciences, Institute of Environmentally Friendly Agriculture, Chonnam National University, Gwangju, South Korea; ^2^Department of Integrated Biological Science, College of Natural Science, Pusan National University, Busan, South Korea; ^3^Forest Insect Pests and Diseases Division, National Institute of Forest Science, Seoul, South Korea

**Keywords:** pine wilt disease, methyl salicylate, systemic acquired resistance, foliar application, qRT-PCR

## Abstract

Pine wilt disease (PWD), caused by the pinewood nematode, is the most destructive disease in pine forest ecosystems worldwide. Extensive research has been done on PWD, but effective disease management is yet to be devised. Generally, plants can resist pathogen attack via a combination of constitutive and inducible defenses. Systemic acquired resistance (SAR) is an inducible defense that occurs by the localized infection of pathogens or treatment with elicitors. To manage PWD by SAR in pine trees, we tested previously known 12 SAR elicitors. Among them, methyl salicylate (MeSA) was found to induce resistance against PWD in *Pinus densiflora* seedlings. In addition, the foliar applications of the dispersible concentrate-type formulation of MeSA (MeSA 20 DC) and the emulsifiable concentrate-type formulation of MeSA (MeSA 20 EC) resulted in significantly reduced PWD in pine seedlings. In the field test using 10-year-old *P. densiflora* trees, MeSA 20 DC showed a 60% decrease in the development of PWD. Also, MeSA 20 EC gave the best results when applied at 0.1 mM concentration 2 and 1 weeks before pinewood nematode (PWN) inoculation in pine seedlings. qRT-PCR analysis confirmed that MeSA induced the expression of defense-related genes, indicating that MeSA can inhibit and delay the migration and reproduction of PWN in pine seedlings by modulating gene expression. These results suggest that foliar application of MeSA could reduce PWD incidence by inducing resistance and provide an economically feasible alternative to trunk-injection agents for PWD management.

## Introduction

Pine wilt disease (PWD), caused by the pinewood nematode (PWN), *Bursaphelenchus xylophilus*, is the most destructive disease in pine forest ecosystems worldwide. PWN can easily migrate within infected trees, but because they cannot move between hosts, they are transmitted through the vector *Monochamus* spp. (Mamiya, 1988; Kim et al., 2021). PWN is fatal to healthy susceptible pine trees, such as *Pinus densiflora*, *P. thunbergii*, and *P. koraiensis* and new susceptible species are being discovered (Yang and Wang, 1989; Li et al., 2020). PWD-infected pine trees initially turn their needles yellowish to reddish-brown, and then subsequently wilt and die within a few months. PWD was first reported in Japan in 1905 (Yano, 1913), after which it gradually reached Europe (Portugal and Spain) as well as East Asian countries such as China, Taiwan, and Korea (Cheng et al., 1983; Tzean and Jan, 1985; Yi et al., 1989; Mota et al., 1999; Abelleira et al., 2011).

The serious threat posed by PWN has led to considerable efforts to control the disease. Disease control mostly relies on use of chemicals such as metam sodium for fumigation of infected trees, aerial spraying of pesticides to control pine sawyer, and trunk injection of abamectin and emamectin benzoate (Kwon et al., 2005; Lee et al., 2009; Korea Forest Service, 2013). However, with the growing awareness regarding environmental conservation, there is now a demand for environmental-friendly control measures. In line with this, research on elucidating resistance mechanisms related to the relationship between pathogens and host pines is being emphasized upon.

Plants can resist pathogen attack through a combination of constitutive and inducible defense mechanisms, which suppress pathogen reproduction and spread (Spoel and Dong, 2012; Vlot et al., 2021). Induced resistance has two forms: systemic acquired resistance (SAR) and induced systemic resistance (ISR). Specifically, SAR is known as long-lasting and broad-spectrum resistance that sets in following a localized infection by a variety of pathogens or treatment with SAR elicitors. The activation of SAR requires transfer of signals from the primarily infected tissue to systemic tissue. Various substances that activate SAR, such as β-aminobutyric acid (BABA), methyl salicylate (MeSA), and azelaic acid (AZA) have recently been reported (Park et al., 2007; Jung et al., 2009; Ji et al., 2015).

MeSA is a naturally occurring compound that is widely distributed in many plants and is classified into a group of plant hormones that play various regulatory roles in plant metabolism (Hayat and Ahmad, 2007). MeSA, a long-distance signaling compound of SAR, is volatilized and transported to the air. Airborne MeSA is absorbed by uninfected distal tissues, converted into SA, after which it eventually activates SAR (Dempsey and Klessig, 2012). Recently, upon application of MeSA, development of disease resistance was reported against bacterial blight caused by *Xanthomonas oryzae* pv. *oryzae* in rice and gray mold caused by *Botrytis cinerea* in grape (Kalaivani et al., 2016; García-Pastor et al., 2020).

It has been demonstrated that MeSA can effectively control PWD by inducing flavonoid biosynthesis through comparative *in vivo* transcriptomics on pine seedlings (Park et al., 2020). However, the establishment of optimal treatment conditions for high efficiency of MeSA and application to the field have not been reported. The purpose of this study was to establish the optimal formulation and processing conditions of MeSA for effectively controlling PWD through *in vivo* assays and field experiments, and to elucidate the mechanisms underlying the effects of MeSA against PWD based on qRT-PCR analysis.

## Materials and Methods

### Chemicals and Formulations

AZA (>98%), azoxystrobin (98.4%), BABA (>98%), γ-aminobutyric acid (GABA, > 99%), glucan (from black yeast), isotianil (ITL, 98.2%), methyl jasmonate (>90%), MeSA (>99%), probenazole (PBZ, 98.5%), salicylic acid (SA, > 99.5%), tiadinil (TDL, 98.9%), and validamycin A (VMA, 64.24%) were purchased from Tokyo Chemical Industry Co., Ltd. (Tokyo, Japan). The four elicitors selected following screening tests (GABA, MeSA, PBZ, and SA) were formulated by Yoosung Chemical R&T Co., Ltd. (Daejeon, South Korea). A soluble concentrate-type formulation of GABA (GABA 20 SL) was prepared by mixing 20% (w/w) GABA, 10% (w/w) polyethylene glycol-400, 5.0% (w/w) sodium lignin sulfonate, 3.0% (w/w) monotridecyl ether (POE9), and 62.0% (w/w) water. A dispersible concentrate-type formulation of MeSA (MeSA 20 DC) was prepared by mixing 20% (w/w) MeSA, 30% (w/w) propylene glycol monomethyl ether, 20% (w/w) propylene glycol, and 20% (w/w) CR-FL3PG (the mixture of triethanolamine, and polyoxyethylene tristyrylphenyl ether phosphate). An emulsifiable concentrate-type formulation of MeSA (MeSA 20 EC) was prepared by mixing 20% (w/w) MeSA, 20% (w/w) propylene glycol monomethyl ether, 20% (w/w) CR-MOC25 (the mixture of ethoxylated castor oil, calcium dodecylbenzenesulfonate, and tristyrylphenol ethoxylates) as an emulsifier, and 40% (w/w) methylated soybean oil (MOS2). A suspension concentrate-type formulation of PBZ (PBZ 20 SC) was prepared by mixing 20% (w/w) PBZ, 8.0% (w/w) propylene glycol, 5.0% (w/w) CR-FL3PG, 0.12% (w/w) xanthan gum, 0.1% (w/w) silicon defoamer, and 66.78% (w/w) water. A soluble concentrate-type formulation of SA (SA 20 SL) was prepared by mixing 20% (w/w) SA, 11.6% (w/w) NaOH (50%), 10% (w/w) polyethylene glycol-400, 5.0% (w/w) sodium lignin sulfonate, 3.0% (w/w) monotridecyl ether (POE9), and 50.4% (w/w) water.

### Nematodes and Plants

The PWN *Bursaphelenchus xylophilus*, which causes PWD, was isolated from infected pine trees and obtained from the National Institute of Forest Research (NIFoS; Seoul, South Korea). PWN was cultured on the mycelia of *Botrytis cinerea* on potato dextrose agar (Difco; Becton, Dickinson and Company, Sparks, MD, United States) at 25°C for 1 week in dark (Maehara and Futai, 2000). PWNs were harvested from the medium using the Baermann funnel method (Baermann, 1917). For the *in vivo* experiment, 3-year-old *Pinus densiflora* and *P. thunbergii* seedlings of 40–50 cm height and 0.5–1.0 cm diameter were obtained from Daelim seedling farm (Okcheon, South Korea) and transplanted into 15-cm diameter pots containing sterilized nursery soil in the greenhouse, which was maintained at an average temperature of 25°C with the relative humidity of 70%.

### Screening of Various Elicitors Against Pine Wilt Disease by Means of Trunk Injection

Ten chemicals (except glucan and ITL) were dissolved in an aqueous solution containing 10% methanol, while glucan and ITL were dissolved in an aqueous solution containing 10% dimethylformamide, at concentrations of 200 and 20 μg/mL. Three-year-old *P. densiflora* seedlings were treated with 100 μL of each solution via trunk injection. A hole was drilled using a 1.8 mm drill bit at a 45° angle in the trunk of a pine seedling, 5 cm above the ground. A 200 μL pipette tip (Axygen Scientific Inc., Union, CA, United States) was inserted into the hole. One hundred microliters of each chemical solution was injected into the pipette tip. The pipette tip was covered with Parafilm^®^ M (Heathrow Scientific, Vernon Hills, IL, United States) to prevent drying, following which a small hole was made in it using a pin. One week later, the treated pine seedlings were inoculated with PWN, as described below. After making a small slit with a surface-sterilized knife, a small piece of absorbent cotton was inserted into the slit, and a water suspension of nematodes (2,000 nematodes in 100 μL) was pipetted onto the absorbent cotton. The slits were then covered with Parafilm^®^ to prevent drying (Kwon et al., 2010). Distilled water containing 10% methanol was used as an untreated control. Three replicates were included in each treatment, and each experiment was performed twice.

### Effects of Four Selected Elicitors Formulations Against Pine Wilt Disease Upon Foliar Application

Formulations of GABA 20 SL, MeSA 20 DC, PBZ 20 SC, and SA 20 SL were dissolved in distilled water at a concentration of 10 mM each, and then 5 mL of each solution was foliar sprayed onto 3-year-old *P. densiflora* seedlings. The treatment was conducted twice at an interval of 1 week. One week after the second treatment, the treated 3-year-old pine seedlings were inoculated with 2,000 PWNs. Distilled water containing 250 μg/mL Tween^®^ 20 was used as an untreated control and five replicates were used. The severity of PWD was assessed according to the wilting and the ration of the brown leaves of the whole plant leaves (Proença et al., 2010; Kim et al., 2019). To evaluate the control value of treatment against *B. xylophilus*, control value was measured using below formula (Kwon et al., 2010), control value (%) = (1-disaease severity of treatment/disease severity of untreated control) × 100.

### *In vitro* Nematicidal Activity of Methyl Salicylate

To confirm whether the effect of MeSA against PWD was caused by direct nematicidal activity, the nematicidal activity of MeSA was evaluated on *B. xylophilus*. After the suspension containing approximately 50 nematodes was added to each well of a 96-well microplate (Becton Dickinson, Franklin Lakes, NJ, United States), treatments with three concentrations of MeSA, 1,000, 333, and 111 μg/mL, were carried out. Sterilized distilled water was used as the negative control. The plates were gently shaken and incubated in a dark plastic box with 100% humidity at an average temperature of 25°C. All experiments were conducted in triplicates and repeated twice. Nematicidal activity was evaluated at 3 days post-treatment, under an optical microscope (Leica DM IL LED; Leica Microsystems CMS GmbH, Wetzlar, Germany). To analyze the nematicidal activity of MeSA against *B. xylophilus*, mortality was converted to percentage mortality and corrected using Abbott’s formula (Abbott, 1925), Mortality (%) = [(Mortality percentage in treatment – Mortality percentage in the negative control)/(100 – Mortality percentage in the negative control)] × 100.

### Evaluation of Disease Control Efficacy of Methyl Salicylate 20 DC Against Pine Wilt Disease Under Field Conditions

The field experiment was performed in a pine forest located in Seongbang-ri, Gonmyeong-myeon, Sacheon-si, Gyeongsangnam-do province, Republic of Korea, in 2017. Ten-year-old *P. densiflora* trees, 3–5 m in height and 8–10 cm in diameter at chest height, were used for the experiment. MeSA 20 DC formulation was prepared in distilled water at a concentration of 10 mM and 500–800 mL for a tree. This solution was foliar sprayed onto 10-year-old *P. densiflora* trees through Sprayer Clover (Model TH-33; Taehwan Co., South Korea). The treatment was conducted twice at an interval of 1 month. One month after the second treatment, the treated pine trees were inoculated with PWN via trunk injection. A hole was drilled using a 10 mm drill bit at a 45° angle into the trunk of a pine tree, 30 cm above the ground. One milliliter of water suspension of nematodes (10,000 nematodes) was pipetted into the hole. Inoculation of PWN was performed on June 2, 2017, and the average temperature was 20°C. After inoculation with PWN, the hole was closed using a cork stopper. Distilled water containing 250 μg/mL Tween^®^ 20 was foliar sprayed as a negative control, while emamectin benzoate 2.15 EC (Affirm, Syngenta Co., Seoul, Korea) was administered via trunk injection as a positive control. Sixteen trees were used in each treatment. The incidence of PWD was assessed in terms of the trees being either alive or dead.

### Selection of Optimal Formulation of Methyl Salicylate Against Pine Wilt Disease in 3-Year-Old *Pinus densiflora* and *Pinus thunbergii* Seedlings

MeSA 20 DC and MeSA 20 EC were formulated by Yoosung Chemical R&T Co., Ltd. To select the optimal formulation of MeSA, *in vivo* assays were performed on *P. densiflora* and *P. thunbergii* seedlings. Each sample was dissolved in distilled water at a concentration of 10 mM, and then 5 mL of these solutions were foliar sprayed onto 3-year-old *P. densiflora* and *P. thunbergii* seedlings. The treatment was conducted twice at an interval of 1 week. One week after the second treatment, the treated seedlings were inoculated with 2,000 PWNs. Distilled water containing 250 μg/mL Tween^®^ 20 was used as a negative control, and emamectin benzoate 20 mg/mL was used as a positive control. Five replicates were included in each treatment group. The severity of PWD was assessed according to the wilting and discolored area of the needles.

### Determination of Optimal Concentration and Application Time of Methyl Salicylate 20 EC Against Pine Wilt Disease in 3-Year-Old *Pinus densiflora* and *Pinus thunbergii* Seedlings

To determine the optimal concentration of MeSA 20 EC, *in vivo* assays were performed at various concentrations of MeSA 20 EC to select the optimal formulation for MeSA. MeSA 20 EC was dissolved in distilled water at concentrations of 10, 0.1, and 0.01 mM and then 5 mL of these solutions were foliar sprayed onto 3-year-old *P. densiflora* and *P. thunbergii* seedlings. The treatment was conducted twice at an interval of 1 week.

For determination of optimal application time for MeSA 20 EC, it was dissolved in distilled water at a concentration of 0.1 mM, which was selected as the optimal concentration, and then foliar sprayed as follows: 1 week before inoculation (WBI) with PWN; 2 and 1 WBI with PWN; 3 and 1 WBI with PWN; 4 and 1 WBI with PWN. In both the experiments aimed at optimization of concentration and application time, the treated seedlings were inoculated with 2,000 PWNs 1 week after the second treatment. Distilled water containing 250 μg/mL Tween^®^ 20 was used as a negative control. Five replicates were included in each treatment group. The severity of PWD was assessed according to the wilting and discolored area of the needles.

### Assessment of the Effect of Methyl Salicylate on the Expression of Defense-Related Genes Using qRT-PCR

Three-year-old *P. densiflora* seedlings were used to analyze the effect of MeSA on expression of defense-related genes. The seedlings were foliar sprayed with MeSA 20 EC (0.1 mM) at an interval of 1 week. For the untreated controls, distilled water containing Tween^®^ 20 (250 mg/L) was used. One week after the second treatment, the treated pine seedlings were inoculated with PWN (2,000 nematodes/100 μL). Three replicates were included in each treatment.

Total RNA was extracted from the pine needles using a hexadecyltrimethylammonium bromide (CTAB)-based extraction buffer (Azevedo et al., 2003). Sampling was performed at 1, 3, and 7 days post-first treatment; 1, 3, and 7 days post-second treatment; and 1, 3, and 7 days post-inoculation with PWN, to confirm the change over time. First, the samples were coarsely ground in liquid nitrogen using a mortar and pestle. The coarsely ground sample (0.5 g) was put into a 2 mL e-tube and completely ground using a tissuelyser (Qiagen Inc., Valencia, CA, United States). The ground sample was mixed with 1 mL extraction buffer containing 0.1 M Tris-HCl (pH 8.0), 30 mM ethylenediaminetetraacetic acid, 2 M NaCl, 2% CTAB, 0.05% spermidine, 2% polyvinylpolypyrrolidone, 2% 2-mercaptoethanol, and 1.5 mg/mL proteinase K. The suspension was incubated at 42°C for 90 min and then extracted with 15 mL chloroform-isoamyl alcohol (24:1). Total RNA was precipitated using 2 M LiCl. Total RNA was further purified using the IQeasy Plus Plant RNA Extraction Mini Kit (iNtRON, Seongnam, South Korea). RNA quality was assessed using a NanoDrop ND-1000 spectrophotometer (NanoDrop Technologies, Wilmington, DE, United States). The cDNA library was synthesized using oligo (Ji et al., 2015) primers and SuperScript IV reverse transcriptase (Invitrogen Inc., Carlsbad, CA, United States), following the manufacturer’s guidelines. The PCR primers used in this study (Table 1; Hirao et al., 2012; Lee et al., 2019) were synthesized by Genotech (Daejeon, South Korea).

**TABLE 1:** Primers used in this study.

Gene	Sequence (5′→3′)	References
PR-1 For	TGCCCCTTCAGGTAAATCGT	Hirao et al., 2012
PR-1 Rev	GCGGGTCGTAGTTGCAGATAA	
PR-2 For	CGACAACATTCGCCCCTTCT	
PR-2 Rev	CTGCAGCGCGGTTTGAATAT	
PR-5 For	GAACCAGTGCCCATACACAGTCT	
PR-5 Rev	CCTGCGGCAACGTTAAAAGTC	
PR-9 For	ACACCACCGTGCTGGACATT	
PR-9 Rev	GTGCGGGAGTCGGTGTAGAG	
ETS For	CGAATGTAATTCCGAAGTTGCA	
ETS Rev	CCATCCCAAACCACCAGTCT	
XET For	TCTGCGCCCCTACTTTTCC	
XET Rev	AGCTGGGCGATTGATCATGT	
PdEIF4A-2 For	AATGCTTGTCCCACCAACAC	Lee et al., 2019
PdEIF4A-2 Rev	AGTGTCAGGCGCTAGTTTTG	

qRT-PCR was performed using iQ™ SYBR Green supermix with a CFX96 Touch™ Real-Time PCR Detection System (Bio-Rad, Hercules, CA, United States). Relative expression levels were calculated using the comparative 2^–ΔΔCT^ method, with *EIF4A-2* as an internal control (Livak and Schmittgen, 2001).

### Statistical Analysis

Statistical analysis was performed to determine significant differences between the groups. All statistical analyses were performed using SPSS statistical analysis software (version 21.0 for Windows; SPSS, IBM Corp., Armonk, NY, United States). Data were evaluated using one-way analysis of variance, and the means of the treatments were determined using the Tukey’s HSD test. Results with *p* < 0.05 were considered statistically significant.

## Results

### Screening of Various Elicitors Against Pine Wilt Disease by Means of Trunk Injection

Twelve elicitors known to induce resistance in various plants were used for *in vivo* screening of PWD in *P. densiflora* seedlings (Table 2). Treatment with the elicitors was carried out via trunk injection to ascertain their effects. Among the 12 candidates, four elicitors (GABA, MeSA, PBZ, and SA) showed more than 75% control values at both the concentrations of 200 and 20 μg/mL (Table 2). Glucan, TDL, and VMA showed control effects at specific concentrations but their effects at the other concentration were not significant.

**TABLE 2:** screening of various elicitors against pine wilt disease by trunk injection into 3-year-old *Pinus densiflora* seedlings.

Treatment	Concentration (μg/mL)	
	200	20
Azelaic acid	+	+
Azoxystrobin	+	+
β-aminobutyric acid	+	−
γ-aminobutyric acid	+++	++
Glucan	+	++
Isotianil	++	+
Methyl jasmonate	+	+
Methyl salicylate	+++	+++
Probenazole	++	+++
Salicylic acid	+++	++
Tiadinil	++	+
Validamycin A	++	+
Control	−	−

*+++, high activity, more than 75% of control value;++, moderate activity, 74–50% of control value; +, low activity, 49–25% of control value; −, no activity, 24–0% of control value.*

### Control Effect of the Four Selected Elicitor Formulations Against Pine Wilt Disease Upon Foliar Application Onto 3-Year-Old *Pinus densiflora* Seedlings

**FIGURE 1:**
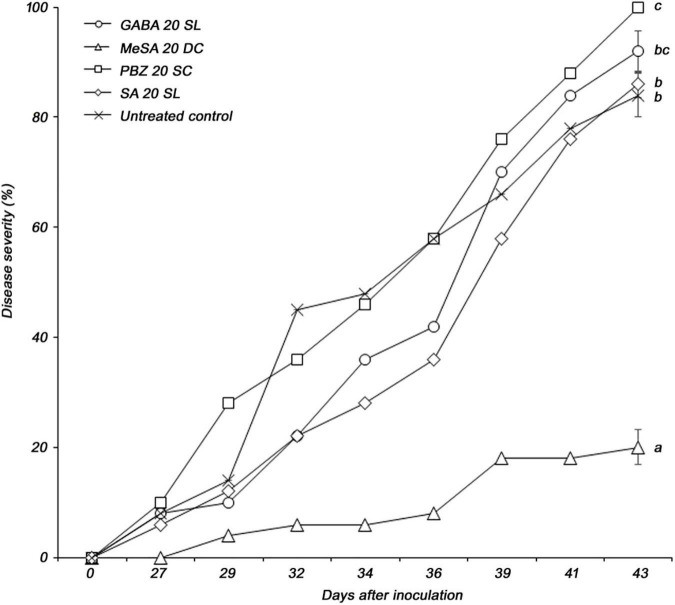
Pine wilt disease severity in 3-year-old *Pinus densiflora* seedlings treated by foliar application with formulations of four elicitors (GABA, γ-aminobutyric acid; MeSA, methyl salicylate; PBZ, probenazole; and SA, salicylic acid). Each value represents the mean ± standard error of five replicates. The different letters indicate significant differences with *p* < 0.05, as calculated using the Tukey’s HSD test.

The four selected elicitors were formulated for stability according to the properties of each chemical. Foliar application was used to control PWD more effectively. Among the four selected formulations, only MeSA 20 DC significantly reduced the severity of PWD in *P. densiflora* seedlings. Disease severity in *P. densiflora* seedlings foliar sprayed with MeSA 20 DC was significantly reduced compared to that in the untreated control. The MeSA 20 DC-treated and -untreated groups showed disease severity of 20 and 84%, respectively, at 43 days post-inoculation with PWN, while the MeSA 20 DC-treated group showed 76.2% control value (Figure 1). GABA 20 SL, PBZ 20 SC, and SA 20 SL did not affect PWD when foliar sprayed. Additionally, when tested against the PWN, *B. xylophilus*, no nematicidal activity of MeSA was observed. MeSA led to 3.5, 1.9, and 0.8% mortality at concentrations of 1,000, 333, and 111 μg/mL, respectively, and showed results comparable to distilled water (mortality: 2.1%) used as a negative control (Supplementary Table 1). On the contrary, emamectin benzoate, which used as a positive control, showed a high mortality of 98.8% even at a very low concentration of 0.11 μg/mL. MeSA leads to approximately 95% mortality at a concentration of 2,000 μg/mL (Kim et al., 2011), but no such activity was observed when it was used at a concentration of 200 μg/mL in the screening test. Therefore, it indirectly suggested that the control effect of MeSA against PWD was due to inducing resistance, not direct nematicidal activity.

### Determination of Optimal Formulation, Concentration, and Application Time of Methyl Salicylate Against Pine Wilt Disease in 3-Year-Old *Pinus densiflora* and *Pinus thunbergii* Seedlings

**FIGURE 2:**
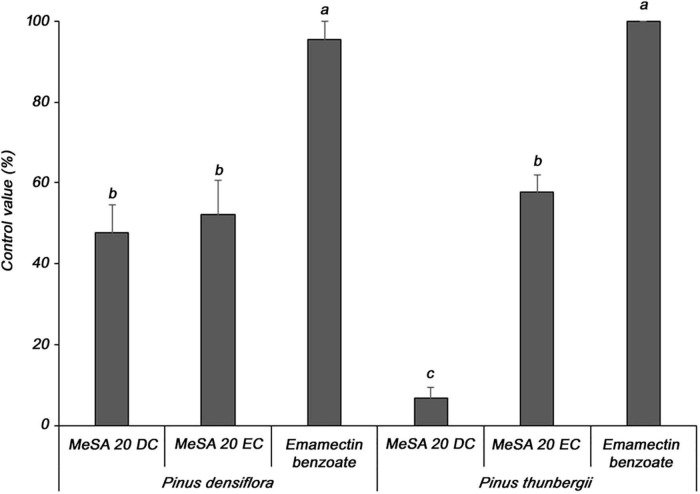
Determination of the optimal formulation of methyl salicylate against pine wilt disease in 3-year-old *Pinus densiflora* and *Pinus thunbergii* seedlings. The formulations of methyl salicylate were treated with 10 mM, and emamectin benzoate was treated with 20 mg/mL. Each value represents the mean ± standard error of five replicates. Different letters indicate significant differences with *p* < 0.05, as calculated using the Tukey’s HSD test.

Two formulations of MeSA were evaluated for their ability to control PWD in *P. densiflora* and *P. thunbergii* seedlings, which are susceptible to PWD. In *P. densiflora*, the MeSA 20 DC and MeSA 20 EC formulations showed 47.7 and 52.3% control values, respectively, compared to the untreated group; moreover, there was no significant difference between the two formulations (Figure 2). In contrast, against *P. thunbergii*, the MeSA 20 DC and MeSA 20 EC formulations showed 6.7 and 57.8% control values, respectively, compared to the untreated group. Furthermore, it was confirmed that the activity of the MeSA 20 DC formulation was significantly reduced. Emamectin benzoate, which was used as a positive control in *P. densiflora* and *P. thunbergii*, showed excellent control values of 95.5 and 100%, respectively. Therefore, MeSA 20 EC was selected as the optimal formulation, and was used for the subsequent experiments.

**FIGURE 3:**
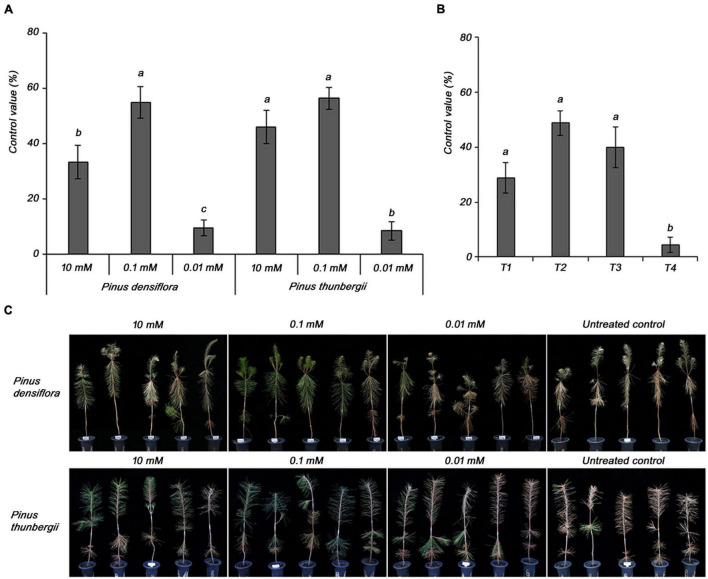
Determination of **(A)** optimal concentration of methyl salicylate 20 EC in 3-year-old *Pinus densiflora* and *Pinus thunbergii* seedlings, and **(B)** application time of 0.1 mM methyl salicylate 20 EC in 3-year-old *Pinus densiflora* seedlings against pine wilt disease. T1, treatment at 1 week before inoculation with pine wood nematode; T2, treatment at 2 and 1 weeks before inoculation with pine wood nematode; T3, treatment at 3 and 1 weeks before inoculation with pine wood nematode; T4, treatment at 4 and 1 week before inoculation with pine wood nematode. Each value represents the mean ± standard error of five replicates. Different letters indicate significant differences with *p* < 0.05, as calculated using the Tukey’s HSD test. **(C)** The representing pictures of *P. densiflora* and *P. thunbergii* after inoculation with PWN with or without MeSA. From 10 to 0.01 mM of MeSA was treated 2 and 1 weeks before PWN inoculation with indicated concentrations.

Subsequently, the optimal concentration of MeSA 20 EC formulation was determined for the effective control of PWD. In *P. densiflora*, the control value was higher (54.8%) at 0.1 mM MeSA 20 EC, a concentration 100-fold lower than the other tested concentration, which was 10 mM (33.3%) (Figures 3A,C). However, 0.01 mM did not control PWD. The results in *P. thunbergii* showed a higher control value at 0.1 mM (56.3%) than at 10 mM (45.8%), similar to the results in *P. densiflora*. Therefore, 0.1 mM was selected as the optimal concentration of the MeSA 20 EC formulation for effectively controlling PWD.

The optimal treatment time was determined using 0.1 mM MeSA 20 EC as the optimal concentration. The control values were 48.9 and 40.0% for T2 and T3, respectively. There was no significant difference between T2 and T3, but considering T4 (4.4%), we inferred that longer treatment interval worsened the efficacy. Therefore, the optimal treatment time was selected as T2 (Figure 3B).

### Evaluation of Disease Control Efficacy of Methyl Salicylate 20 DC Against Pine Wilt Disease Under Field Conditions

**FIGURE 4:**
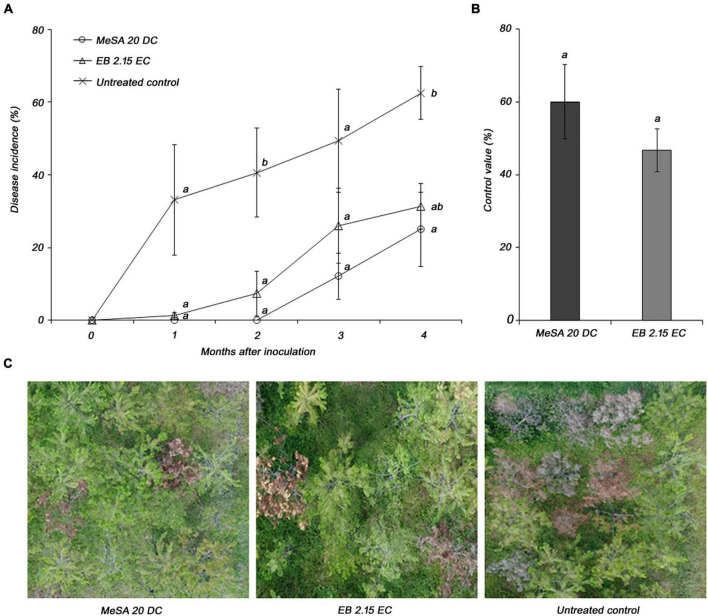
Evaluation of disease control efficacy of methyl salicylate 20 DC (MeSA 20 DC) against pine wilt disease upon foliar application onto 10-year-old *Pinus densiflora* under field conditions. Emamectin benzoate 2.15 EC (EB 2.15 EC) was used as positive control and applied via trunk injection. Each pine tree was inoculated with 10,000 juveniles of pine wood nematode. **(A)** Disease severity after inoculation of pine wood nematode. Each value represents the mean ± standard error of four replicates with each replicate consist of four trees. The different letters indicate significant differences with *p* < 0.05, as calculated using the Tukey’s HSD test. **(B)** Disease control efficacy at 4 months post-inoculation with pinewood nematode. **(C)** The pictures of pine trees treated with MeSA 20 DC, EB 2.15 EC, and Tween^®^ 20 (untreated control).

The disease control efficacy of MeSA 20 DC formulation was evaluated under field conditions using 10-year-old *P. densiflora*. The disease incidences in response to treatment with MeSA 20 DC and control were 25 and 62.5%, respectively, at 4 months post-inoculation (Figure 4A). In addition, MeSA 20 DC and emamectin benzoate 2.15 EC, which was used as positive controls, showed control values of 60 and 46.7%, respectively, compared to the untreated control. There was no significant difference between MeSA and emamectin benzoate (Figure 4B). The pictures taken using drone showed distinct wilting brownish between MeSA treatment and untreated control (Figure 4C). Therefore, based on the results of the field tests, we confirmed that MeSA can effectively control PWD in adult trees as well as 3-year-old seedlings.

### Assessment of the Effect of Methyl Salicylate on the Expression of Defense-Related Genes Using qRT-PCR

**FIGURE 5:**
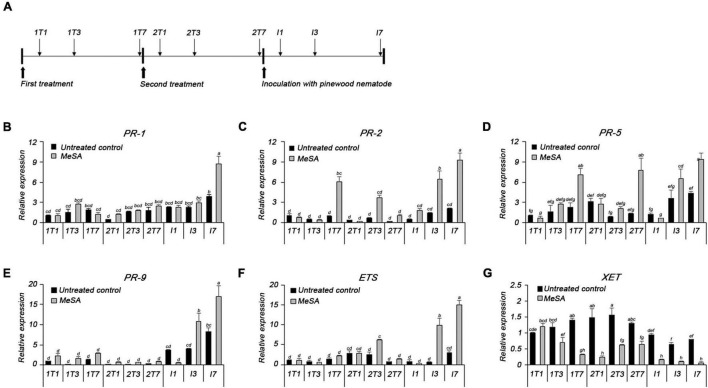
**(A)** The plan of methyl salicylate treatment, pinewood nematode inoculation, and sampling for qRT-PCR analysis. qRT-PCR analysis of transcripts of **(B)** pathogenesis-related protein 1 (*PR-1*), **(C)** pathogenesis-related protein 2 (*PR-2*), **(D)** pathogenesis-related protein 5 (*PR-5*), **(E)** pathogenesis-related protein 9 (*PR-9*), **(F)** extensin (*ETS*), and **(G)** xyloglucan endotransglycosylase (*XET*).

The expression of defense-related genes was analyzed using qRT-PCR following treatment with PWN, MeSA, and both PWN and MeSA (Figure 5A). Expressions of *PR-1*, *PR-2*, *PR-5*, and *ETS* were modulated upon PWN inoculation or MeSA spraying. The relative expression level of the *PR-1* was similar to or slightly higher in the MeSA-treated group than in the untreated group; however, the MeSA-treated group showed a 2.2-fold higher expression level than the untreated group at 7 days post-inoculation with PWN (I7) (Figure 5B). The relative expression level of the *PR-2* was 6.2- and 5.7-fold higher than that of the untreated control group at 7 days post-first treatment (1T7) and 3 days post-second treatment with MeSA (2T3), respectively. Post-inoculation with PWN, the expression of *PR-2* was consistently higher than that of the untreated group. The expression levels of *PR-2* in the untreated group also increased over time, but the MeSA-treated group showed a much greater increase; in particular, it displayed 4.4- and 4.5-fold higher upregulation than the untreated group at 3 and 7 days post-inoculation with PWN (I3 and I7), respectively (Figure 5C). The relative expression levels of *PR-5* was 3.1- and 6-fold higher in the MeSA-treated group than in the untreated group at 7 days post-first and -second treatment with MeSA (1T7 and 2T7), and 1.8- and 2.2-fold higher, respectively, at 3 and 7 days post-inoculation with PWN (Figure 5D). *PR-1*, *PR-2*, and *PR-5*, which are the markers of salicylic acid-dependent SAR, showed similar patterns of upregulation as the untreated groups, post-treatment with MeSA and -PWN inoculation. Upon treatment with PWN or MeSA, *PR-9* expression was induced 5- or 2-fold, respectively, as compared to the untreated condition, at all the time points monitored. Upon treatment with both PWN and MeSA, however, *PR-9* expression was induced up to 15-fold as compared to that in the normal condition, and 2–3-fold that in the PWN inoculation only condition (Figure 5E). These results demonstrated the boosting effect of MeSA treatment, presenting a strong evidence that MeSA treatment and PWN target the same plant defense system.

The MeSA-treated group displayed 2.5-fold higher expression of *ETS*, a cell wall-related gene, than the untreated group, at 3 days post-second treatment with MeSA (2T3). The expression level of *ETS* markedly increased 17.1- and 5.1-fold, respectively, compared to the untreated group, at 3 and 7 days post-inoculation with PWN (I3 and I7) (Figure 5F). Similarly, the *PR-9* expression was also induced at I3 and I7, as compared to the untreated group (2.7- and 2.1-fold, respectively). On the contrary, expression of *XET*, which loosens the cell wall, was downregulated as compared to the untreated group, except at 1 day post-first treatment with MeSA, and was expressed at a very low level post-inoculation with PWN (Figure 5G).

Based on the results of the gene expression test, we concluded that treatment with MeSA via foliar spraying induced SAR in pine trees, including the PWN-related defense system. In addition, MeSA application strengthened the cell wall structure by upregulating *ETS* or downregulating *XET* expression, thereby increasing pine tree defense against PWN.

## Discussion

The control of PWD is a challenging process particularly in large scale forests where trunk injection with nematicides is hardly applicable and would require rigorous effort. In addition, spraying insecticides against the *Monochamus* vector, that is among the main methods used for conventional PWD management, was shown to have a limited effect in hindering PWD spread and might result in emergence of insect resistance along with encompassing a great deal of environmental burden and adverse effects on human and other non-target species (Ikenaka et al., 2019; Jung et al., 2021). Induction of resistance using chemical elicitors represent a promising alternative that is sustainable and eco-friendly as there is no direct nematicidal activity or effect on other non-target species (Oka et al., 2000). However, utilization of induced resistance elicitors require evaluation and accurate setting up of the effective formulation, concentration, and time of foliar spray application. In this study, four SAR elicitors, including MeSA, were selected among 12 candidates known to induce resistance in various plants, based on *in vivo* screening via trunk injection into 3-year-old *P. densiflora* seedlings. The stability and effectiveness of the four selected resistance elicitors were assessed and MeSA was selected post*-in vivo* testing of its potency upon application as a foliar spray. MeSA is a volatile long-range signaling compound produced by many plants for induction of SAR and may activate resistance in neighboring plants or in the healthy tissues of the infected plant. MeSA in the air is absorbed by the uninfected distal tissue and converted to SA for the establishment of acquired resistance (Shulaev et al., 1997). Signaling in plants by volatile compounds such as MeSA can overcome the spatial and temporal limitations of the vascular system (Heil and Ton, 2008). The timing of defense responses is crucial and can mark the difference between whether the plant overcomes or succumbs to the pathogen infection.

Two types of MeSA formulations (MeSA 20 DC and MeSA 20 EC) were tested for foliar spray application in this study. Unlike *P. densiflora*, treatment with MeSA 20 DC was ineffective in controlling PWD when tested on *P. thunbergii* seedlings. Although both *P. densiflora* and *P. thunbergii* belong to the same genus *Pinus*, their leaves are histologically different, as *P. thunbergii* has a thicker epidermis and stronger subcutaneous tissue than *P. densiflora* (Shaw, 1914). To overcome this difference, a new formulation, MeSA 20 EC, was prepared. Upon comparing the two formulations, MeSA 20 EC showed better control efficiency in both *P. densiflora* and *P. thunbergii* seedlings (52.3 and 57.8%, respectively), while MeSA 20 DC showed a similar effect (47.7%) to MeSA 20 EC in *P. densiflora*, but not in *P. thunbergii* (6.7%). Unlike DC, EC uses methylated soy bean oil (MSO2) as the solvent with a high flash point, thus resulting in a lower volatility of MeSA, allowing for prolonged exposure on the leaves, thus improving its efficiency on *P. thunbergii*. Therefore, the MeSA 20 EC formulation was selected as the optimal formulation.

Resistance inducing elicitors generally show maximum efficiency at specific concentrations, depending on the plant, unlike chemicals that have direct concentration-dependent antimicrobial activity. For example, SA showed the highest effect at a concentration of 1.5 mM against *Fusarium oxysporum* in tomato and at a concentration of 0.5 mM against *Ralstonia solanacearum* in pepper (Ojha and Chatterjee, 2012; Chandrasekhar et al., 2017). We tested several concentrations of MeSA 20 EC formulation to determine the optimal concentration, which was eventually selected as the optimal formulation for treating PWD. The highest control efficiency was observed at MeSA concentration of 0.1 mM in both *P. densiflora* and *P. thunbergii* (54.8 and 56.3%, respectively). Using this optimum concentration of MeSA 20 EC, the optimal treatment time was tested and confirmed the control values of 48.9 and 40.0% at T2 and T3, respectively. There was no significant difference between T2 and T3, but considering the effect at T4 (4.4%), we inferred that the longer treatment interval worsened the efficacy; thus, T2 was selected as the optimal treatment time. Based on these results, it was established that treatment with MeSA 20 EC formulation at a concentration of 0.1 mM at 2 and 1 week before inoculation of PWN (T2) were the optimal formulation and processing conditions for MeSA, respectively, to achieve effective control against PWD.

Field experiments on 10-year-old *P. densiflora* have also confirmed the efficiency of MeSA foliar treatment against PWD as the disease incidence of treated trees were significantly reduced to levels comparable to those achieved with trunk injection with Emamectin benzoate. However, since we have established that 0.1 mM of MeSA EC is the optimal treatment condition through the optimized greenhouse test of MeSA, additional field tests according to the optimal treatment conditions are required, and a better effect can be expected.

Our previous studies reported genome-wide analyses of pine trees treated with MeSA against the nematode *B. xylophilus* (Park et al., 2020). The application of the MeSA, effective in terms of reducing PWD severity, mainly induce genes involved in systems for protection from ROS damage as well as genes encoding flavonoid biosynthesis. Flavonoids not only contribute to a neutralizing ROS (Agati et al., 2012), but affect the fitness of nematodes at different life stages (Chin et al., 2018) based on network and gene ontology (GO) analyze. In this study, we focused on expression analyze of defense-related genes. It has been reported that the transcript levels of defense-related gene expression are different between susceptible and resistance pine trees and the inoculation with PWN further increase such difference in gene expression levels (Hirao et al., 2012). To elucidate the mechanism underlying the observed control value of MeSA against PWD, qRT-PCR analysis was performed in this study using *P. densiflora* leaves. When MeSA was applied to pine leaves, *PR-1*, *PR-2*, and *PR-5* expressions were significantly upregulated compared to the untreated group, before and after inoculation with PWN. *PR-1*, *PR-2*, and *PR-5* are commonly used as marker genes for SAR induction (Molinari et al., 2014). Recent studies have reported that upregulation of *PR-1*, *PR-2*, and *PR-5* expression significantly inhibits infection of beet-cyst (*Heterodera schachtii*) and root-knot (*Meloidogyne incognita*) nematodes in *Arabidopsis thaliana* and tomato (Uehara et al., 2010; Hamamouch et al., 2011). Furthermore, it has been reported that the difference between whether a plant is susceptible or resistant depends on the differences in the timing and magnitude of defense response (Tao et al., 2003). Therefore, the optimum timing and high expression of the *PR* may contribute to the effective inhibition of PWD development.

Upon MeSA treatment, expressions of cell wall-related genes, *PR-9* and *ETS*, were upregulated, while that of *XET* was downregulated. Extensin, encoded by the *ETS*, is believed to play an important part in cell wall biosynthesis in plants (Cannon et al., 2008). Extensin plays an essential role in biotic and abiotic stress responses by being involved in maintaining the strength of the cell wall (Deepak et al., 2010). In addition, peroxidase encoded by the *PR-9* strengthens the cell wall by catalyzing lignification and enhancing resistance against various pathogens (Van Loon et al., 2006). The *XET* regulates the strength of the cell wall by being involved in xyloglucan, which supports the load between the cellulose microfibers in the cell wall (Uozu et al., 2000; Vissenberg et al., 2000). Strengthening of the cell wall is a very important factor during the early stages of plant defense. In a study comparing histological changes in susceptible and resistant *P. thunbergii*, the resistant *P. thunbergii* effectively inhibited the initial migration of PWN by strengthening the cell wall via lignification (Kusumoto et al., 2014). In addition, migration and reproduction of PWN are important factors that determine the survival of pine trees (Nakajima et al., 2019). Therefore, MeSA is believed to be involved in a control mechanism against PWD by rapidly inducing the expression of defense-related genes in pine trees prior to infection, promptly responding to invasion of PWN, and delaying migration and reproduction of PWN.

Overall, this study provides evidence on the effective control activity of foliar spray with MeSA against PWD under greenhouse and field conditions as possible alternative to other pesticide control methods. The mechanism of control was indicated as induction of resistance by the observed significant changes in the expression of defense-related genes without direct nematicidal activity. The optimum formulation, concentration and treatment time were adjusted for improvement of the control activity. The provided results represent an important step toward the establishment of effective eco-friendly and applicable control method for PWD under field conditions.

## Conclusion

MeSA was selected from 12 candidate elicitors via *in vivo* screening using *P. densiflora* seedlings. MeSA 20 DC formulation showed a control efficacy of 60% in field tests using 10-year-old *P. densiflora*. The optimal formulation and processing conditions for treatment were established as 0.1 mM MeSA 20 EC at 2 and 1 WBI with PWN. In addition, qRT-PCR analysis showed that MeSA induced elevated expression of various defense-related genes in pine leaves. The high expression of various plant defense-related and cell wall-related genes suggests that PWD can be controlled by inhibiting the migration and reproduction of PWN. Collectively, these results suggest that foliar application of MeSA may reduce the incidence of PWD by inducing resistance, thereby providing an economically feasible alternative to trunk-injection agents for the management of PWD.

## Data Availability Statement

The original contributions presented in the study are included in the article/Supplementary Material, further inquiries can be directed to the corresponding author/s.

## Author Contributions

Y-SS and J-CK conceived the study. HJ, AP, MS, NK, GH, and JK performed the experiments. HJ, AP, Y-SS, and J-CK analyzed the data. HJ wrote the manuscript. AP, Y-SS, YK, and J-CK reviewed and edited the manuscript. J-CK acquired the funds. All authors have read and agreed to the published version of the manuscript.

## Conflict of Interest

The authors declare that the research was conducted in the absence of any commercial or financial relationships that could be construed as a potential conflict of interest.

## Publisher’s Note

All claims expressed in this article are solely those of the authors and do not necessarily represent those of their affiliated organizations, or those of the publisher, the editors and the reviewers. Any product that may be evaluated in this article, or claim that may be made by its manufacturer, is not guaranteed or endorsed by the publisher.
