# Supplementary material for: Systemic Acquired Resistance-Mediated Control of Pine Wilt Disease by Foliar Application With Methyl Salicylate
Source: Front Plant Sci. 2022 Jan 5;12:812414. doi: 10.3389/fpls.2021.812414 (PMC8767056; doi:10.3389/fpls.2021.812414)
Supplement: Supplementary file 1 [file Table_1.DOCX]

**Supplementary Table 1** *In vitro* nematicidal activity of methyl salicylate against pine wood nematode, *Bursaphelenchus xylophilus*

| Chemical | Concentration (μg/mL) | Mortality (%) |
| --- | --- | --- |
| Methyl salicylate | 1000 | 3.5±1.8 b |
|  | 333 | 1.9±1.8 b |
|  | 111 | 0.8±1.3 b |
| Emamectin  benzoate | 1 | 100.0±0.0 a |
|  | 0.33 | 100.0±0.0 a |
|  | 0.11 | 98.8±2.1 a |
| Control (H_2_O) | - | 2.1± 2.4 b |

Each value represents the mean ± standard error of three replicates. The different letters indicate significant differences with p < 0.05, as calculated using Duncan’s multiple range test.
